# Molecular and ionic-scale chemical mechanisms behind the role of nitrocyl group in the electrochemical removal of heavy metals from sludge

**DOI:** 10.1038/srep31828

**Published:** 2016-08-23

**Authors:** S. W. Hasan, I. Ahmed, A. A. Housani, A. Giwa

**Affiliations:** 1 Department of Chemical and Environmental Engineering, Masdar Institute of Science and Technology, P.O. Box 54224, Abu Dhabi, United Arab Emirates

## Abstract

The chemical basis for improved removal rates of toxic heavy metals such as Zn and Cu from wastewater secondary sludge has been demonstrated in this study. Instead of using excess corrosive chemicals as the source of free nitrous acid (FNA) for improved solubility of heavy metals in the sludge (in order to enhance electrokinetics), an optimized use of aqua regia has been proposed as an alternative. Fragments of nitrocyl group originated from aqua regia are responsible for the disruption of biogenic mixed liquor volatile suspended solids (MLVSS) and this disruption resulted in enhanced removal of exposed and oxidized metal ions. A diversity of nitric oxide (NO), peroxy nitrous acid, and peroxy nitroso group are expected to be introduced in the mixed liquor by the aqua regia for enhanced electrochemical treatment. The effects of pectin as a post treatment on the Zn removal from sludge were also presented for the first time. Results revealed 63.6% Cu and 93.7% Zn removal efficiencies, as compared to 49% Cu and 74% Zn removal efficiencies reported in a recent study. Also, 93.3% reduction of time-to-filter (TTF), and 95 mL/g of sludge volume index (SVI) were reported. The total operating cost obtained was USD 1.972/wet ton.

Surging population growth rate and swiftly expanding city lifestyle has turned the living planet into a reservoir of urban waste biosolids. Environmentalists are joining efforts to enable 3R (recycle, reduce, and reuse) technologies for sustainable and economical waste treatment technologies^1^. For example, on average, 26,000 tons of biosolids are generated yearly from urban wastewater treatment plants in the City of Abu Dhabi, United Arab Emirates (UAE); yet these biosolids do not meet local regulatory standards and are landfilled. Landfilling imposes extra socioeconomic cost due to transportation and land space requirements^1^,^2^. Apart from economic burden, excessive exposure of biosolids in UAE to certain metals is a persistent threat to its very limited fauna and flora. These toxic metals can be stored in living tissues through food chain causing severe health and environmental disorders^3^. Although some of these metals (e.g. Cu, Zn, Co, Cd and Fe) are essential elements for the activation of some enzymes in human cells, intolerable exposure levels ranging from 100–500 mg/d can be toxic and even carcinogenic to humans^4^,^5^. Excessive amounts of these metals in the ecosystem threaten soil ecology, agricultural production, and groundwater safety^3^. Zn ions have been associated with ability to replace magnesium (Mg) ions in chlorophyll molecules which can damage DNA molecules by oxidative stress^5^. Therefore, the World Health Organization (WHO) has pegged the tolerable concentration of Zn in drinking water to a low value of 5.0 mg/L^6^. That being said, the removal of heavy metals from secondary sludge/biosolids by an economical and greener approach is indispensable. It is desperately needed to convert huge toxic waste liability into valuable resource for sustainable agriculture and aquaculture^2^.

Various technologies have been tested such as adsorption/biosorption^7^,^8^,^9^, chemical sequestration (through sequential extraction, leaching, or use of chelating agent)^7^,^9^,^10^, electrokinetics^6^,^11^, and acidified sludge treatment^12^,^13^. Electrokinetic (EK) treatment has been a viable waste sludge treatment method because of its ability to remove heavy metals^11^,^14^, break down recalcitrant colloids and organics^15^, reduce soluble microbial products^16^, and enhance sludge physiochemical properties^6^,^17^. In EK, heavy metals are either removed by (a) flocculation and precipitation induced by the formation of coagulants through the hydrolysis of released anodic ions under the influence of electric field^18^,^19^, (b) migrated toward the oppositely charged electrodes where they are removed via electrophoresis and electrodeposition^20^, or (c) removed via floatation enabled by gas bubbles produced from or added to the process^21^. However, EK faces the following three constraints: (a) utilizing high electrical energy for optimum release of electro-coagulants may result in high electrical energy consumption and operation cost^18^; (b) biosolids do not have high electrical conductivity (EC) required to disperse and carry forward electric charges during EK treatment^22^; and (c) heavy metal removal efficiency in EK process is aided by low sludge pH which enhances the solubility of metals. However, most EK processes occur at high pH because of the formation of hydroxides from the hydrolysis of released anodic ions^23^.

Some techniques have been adopted to resolve these constraints. These techniques include: (a) use of low electrical field^6^,^16^,^24^; (b) use of electrochemical process coupled with chelating and leaching agent; and (c) use of acidified sludge to reduce pH and enhance solubility^12^. Among these, bioleaching - a process where sulfide form of metal is oxidized to sulfuric acid through microbial activity - has been found useful in decreasing the pH and solubilizing the metal^25^. Another strategy is to apply an optimized efficient electrokinetic treatment followed by post treatment via organic acids or chelating agents to remove heavy metals from sewage sludge. These agents offer good coordination ability to the metal ions at wide pH range. For example, EDTA has been used because of its strong chelation with alkaline-earth and heavy metals in sewage sludge or soils samples^9^,^26^. Meanwhile, low biodegradability and possibility to persist in the environment has restricted the use of EDTA for large-scale sludge treatment. In addition, advanced oxidation method using hydrogen peroxide (H_2_O_2_) can solubilize the bonded metals but this method is very expensive because of quantitative dose prerequisites such as >10 g/kg dry solids^27^. However none of the above methods could offer a viable solution mainly because of concerns over efficiency, rate of removal, as well as inconsistent activity^25^.

A low electric field treatment activated by low pH has been used in this study. The kinetics of this low-strength EK treatment was improved through the use of aqua regia. Aqua regia contains nitrite which gives rise to free nitrous acid (FNA, HNO_2_) that can increase biodegradability (up to 90%), disrupt extracellular polymeric substances (EPS) and microbial cells, and reduce sludge particle size by breaking down EPS^13^,^28^,^29^. More importantly for this study, FNA can disrupt the organically bounded Zn and Cu trapped in EPS^12^. Biological materials are known for their potential to adsorb heavy metals^30^. EPS have been reported to serve as harbor for toxic metals in municipal waste sludge; therefore the ability of FNA to disrupt EPS and microbial cells would enhance the exposure of toxic metals residing in EPS to electric field. Improvements in the exposure of toxic metals to electric field would ultimately ensure the optimization of electrical energy requirement. None of the previous reports have explained the molecular dynamics relating the influence of precursor of nitrite and its derivatives on EK treatment. This explanation has been presented in this paper. The potential use of sludge as land fertilizer will result in USD 2.83 million (AED 10.4 million) per year as revenue, if it is sold at USD 54.4 (AED 200) per ton^31^. Heavy metal concentration is the major limiting factor hindering sludge reuse and recycling to agricultural land^32^. Therefore, there is a need for a cost-effective treatment method that is capable of removing heavy metals from wastewater secondary sludge. In the present methodology, focus has been directed towards the removal of Zn and Cu because of the very hazardous nature of these heavy metals. The molecular and ionic-scale chemical mechanisms behind the fundamental role of nitrite and their analogous nitro ionic species in the electrochemical removal of heavy metals (Zn and Cu) from wastewater secondary sludge have been provided.

In order to further improve the overall quality of the sludge, a biogenic chelating agent has been used to post-treat the sludge after the low strength acidified EK process. This agent is a cation-capturing organic extract from citrus fruit peel waste known as pectin. Pectin is used as a low cost flocculant and produces harmless intermediates or degradation derivatives after treatment^33^,^34^. Pectin from citrus peels is physically stable, exhibits a particularly high degree of metal uptake^35^, and is also suitable for coagulating residual anodic ions (e.g. Al^3+^ or Fe^3+^) in sludge after EK treatment^33^. However, all these properties of pectin have never been examined and evaluated in solid waste treatment. The effects of pectin post-treatment on physicochemical characteristics of the wastewater secondary sludge such as particle size distribution (PSD), sludge volume index (SVI), dewaterability (time-to-filter, TTF), mixed liquor suspended solids (MLSS), mixed liquor volatile suspended solids (MLVSS), and supernatant turbidity have been explored. Consequently, the overall goal of this study was to optimize the removal of Zn and Cu via low DC voltage EK treatment, minimal chemical additives and relatively greener post-treatment in a very simple engineering design which would result in a low energy consumption wastewater secondary sludge detoxification process.

## Experimental (Materials and Methods)

**Figure 1:**
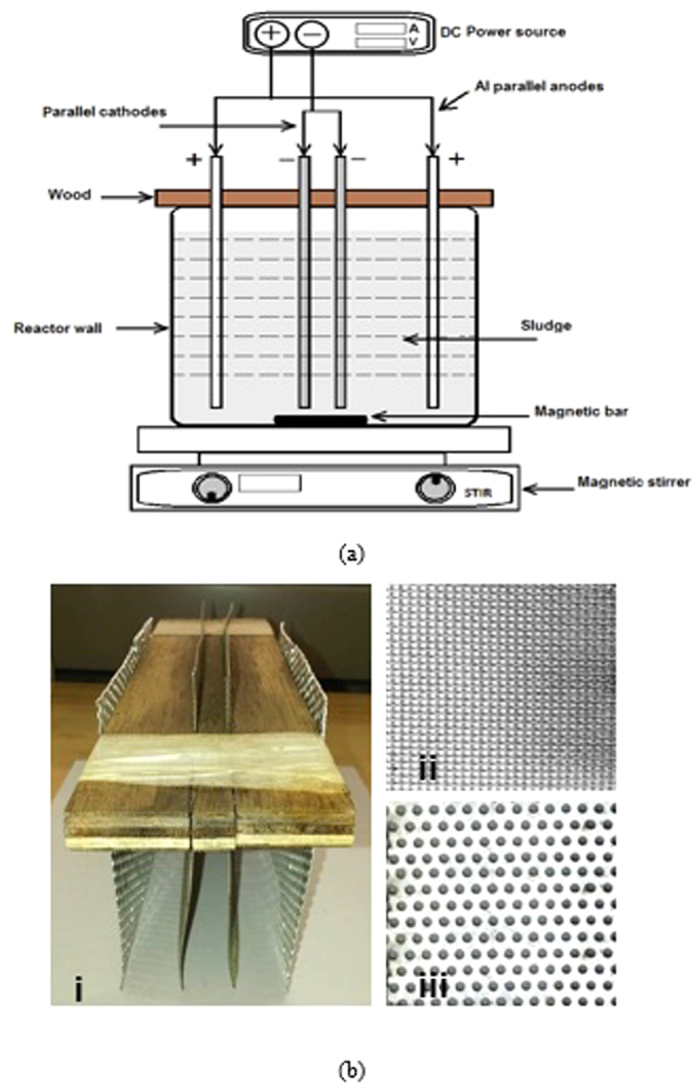
Experimental set-up for EK treatment of waste sludge. (**a**) Lab scale reactor configuration consisting of activated sludge and electrodes. (**b**) (i) Electrodes’ configuration, (ii) stainless steel cathode, and (iii) aluminum anode.

The EK set up consists of a rectangular reactor (Fig. 1a) made up of polyethylene charged with fresh activated sludge obtained from Masdar City membrane bioreactor (MBR) wastewater treatment plant. The anode and cathode were made up of aluminum and stainless steel perforated metal sheets, respectively. These electrodes were arranged in a sequence of anode-cathode-cathode-anode (abbreviated as *A-C-C-A*) and connected to a DC power supply (Fig. 1b). The effective volume of the reactor was 2.5 L, with dimensions of 20 cm × 13 cm × 10 cm. The distance between each anode and cathode was 3 cm while the distance between the cathodes was kept at 1 cm. An effective anode area of 86.88 cm^2^ was used and a distance of 1.5 cm was maintained between the reactor bottom and electrodes for thorough mixing.

The waste sludge sample has been kept in the refrigerator at 4 °C and initial analyses were carried out immediately. The characteristics of the initial wastewater secondary sludge are provided in Table 1. Measurements were carried out in duplicates and mean values were reported. The experiments were designed to achieve the national environment quality standards for biosolids in Abu Dhabi, as shown in Table 2. Zn and Cu were selected as the representative model metals found in biosolid/secondary waste sludge of the UAE. The presented study can be categorized into four phases of treatment as explained in the subsequent sections.

### Optimization of EK process

Experimental work of EK treatment was decided based on orthogonal design of experiments. Three current density of 20, 60 and 100 A/m^2^, and three residence time of 30, 60 and 120 min were fed into the design of experiment tool using Minitab software resulting in a total of 9 experiments to be conducted. The range of current density and treatment time was selected according to our previous research studies to ensure minimum exposure of the sludge to electric field and optimize electrical energy consumption^36^. Current density and residence time were varied in order to optimize the EK process for Cu and Zn removal and improve other physicochemical properties of sludge. A well-mixed sample was taken after each experimental run. The analytical methods used for measurement of physicochemical properties of each sludge sample are summarized in Table 3.

### Addition of chemical conditioners at optimized EK process

The selected optimum current density and sludge residence time was then applied for the EK treatment of the initial sludge. Acidic conditioners plus chelating agent including HNO_3_, EDTA + H_2_SO_4_, aqua regia, and aqua regia + H_2_SO_4_ at pH range of 3–6 were added during this EK treatment. The acid/acid mixtures were used so that low pH values can be maintained with low volumes of acid. The specific conditions used were: HNO_3_ at pH 3, 4 and 6; aqua regia at pH 3; EDTA + H_2_SO_4_ at pH 3; and aqua regia + H_2_SO_4_ at pH 3. To ensure proper mixing of the conditioners in the sludge, the electrodes were first removed from the reactor; then the chemical conditioners were added while the magnetic stirrer was used to ensure homogeneity. After that, pH was measured before placing the electrodes back into the reactor. The concentrations of chemical conditioners used are provided in the Table 4.

### Variation of residence time at optimized acidified EK process

At the optimum current density and selected acidic conditioners, EK residence time was increased to study the influence of residence time on EK treatment efficiency under acidic condition. The optimal case from the addition of chemical conditioners was selected and kept constant but EK residence time was increased to investigate the effect of residence time on the treated sludge properties.

### Investigation of physicochemical characteristics of treated sludge and post-treatment

**Figure 2:**
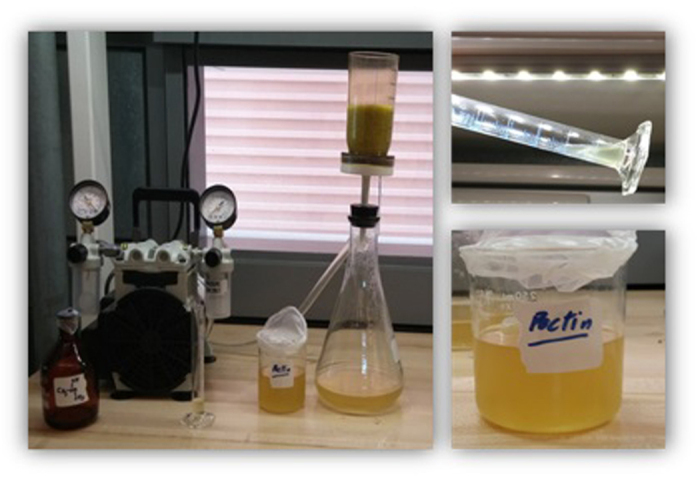
Experimental set-up used for single step green and facile extraction of pectin (left); a qualitative test shows presence of active pectin as white gelatinous shining phase in the bottom of tilted beaker (right).

At the optimized current density and residence time, the physicochemical characteristics of treated sludge samples obtained from the variation of residence time at optimized acidified EK process were determined. Post-treatment was carried out using pectin in order to enhance the coagulation and flocculation of target toxic heavy metal in secondary sludge. This pectin was extracted from the peels of citrus fruit as an attempt to recycle the fruit waste and convert it into an added value product such as a natural and green coagulant^35^. Fresh orange peels were washed adequately with deionized water to remove the extraneous materials. The oranges were peeled off with a focus on the thick white membranous part of the peel as they contain high concentrations of pectin. Then the orange peels were cut into narrow strips and dried at 38 °C in MEMMERT UF55 oven until they were dried completely and reached a constant weight. The dried orange peels were then grounded and sieved with 0.5 mm sieve. 50 mL of formic acid was mixed with 10 g of the dried material and the mixture was kept for 24 h at pH 1 and filtered using Whatmann fritted glass filtration set under vacuum. The obtained filtrate extract contained biocoagulant pectin solution whereas residue could be reprocessed again. To test the presence of pectin qualitatively, three drops of isopropyl alcohol were added to 4 mL of pectin at pH of 1 in a test tube and mixed well. Appearance of shining jelly confirms pectin in the extract. Figure 2 shows the pectin extraction process.

The acidified sludge sample at the selected optimum operating conditions was washed with pectin solution that consisted of 50% pectin at pH 2 and 50% deionized water (ratio 1:1). The sludge was washed again with deionized water in order to remove all the pectin from the sludge till filtrate was at neutral pH. The treated sample was then dried in the oven and the dried sample was prepared for Niton XL3t X-ray fluorescence (XRF) for heavy metal analysis in dry solid phase.

## Results and Discussion

### Optimization of EK conditions for heavy metal removal

**Figure 3:**
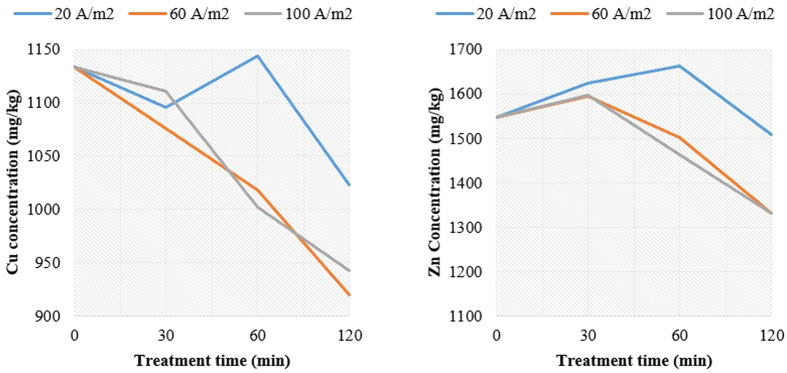
EK removal of Cu (left) and Zn (right) from waste sludge.

Cu concentration in the electro kinetically-treated sludge slurry decreased as the residence time increased (Fig. 3). The applied current density of 60 A/m^2^ was found to offer the best results as Cu concentration decreased from 1133 mg/kg for raw sludge to 920 mg/kg for electrokinetically-treated sludge after 120 min. The current density of 20 A/m^2^ gave the smallest impact with only 9.7% reduction in Cu concentration, as compared with 60 and 100 A/m^2^ which achieved a reduction of 18.8 and 16.9%, respectively after 120 min. 60 A/m^2^ was found to be the optimum current density for Cu, which is close to a reported value of 50 A/m^2^ in the literature^37^. In addition, Zn concentration increased initially from 1548 mg/kg during the first 30 min of operation for all current densities, and then started to decrease at 60 and 100 A/m^2^ to about 1332 mg/kg of Zn after 120 min.

**Figure 4:**
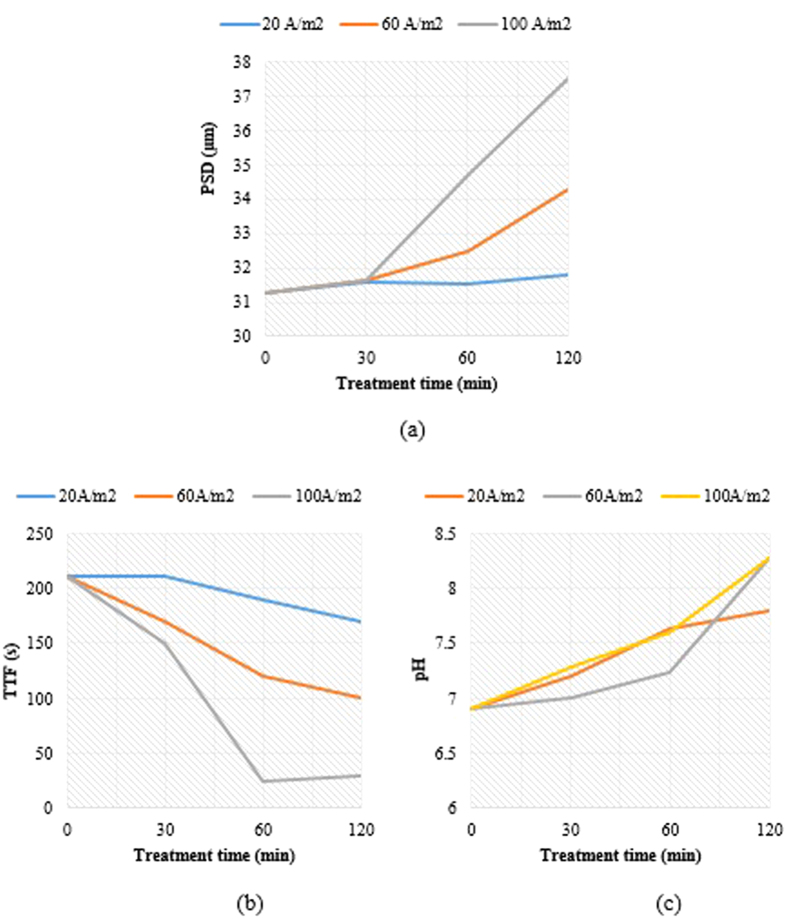
Influence of EK on the physicochemical properties of sludge. (**a**) Effect of EK on sludge mean PSD, (**b**) TTF, and (**c**) pH.

The observed increase in Zn concentration beyond the initial concentration could be attributed to the fact that steel bars or mesh (i.e. cathode material in our experiments) contain Zn through which when exposed to moisture; they are protected by the sacrificial loss of zinc in the vicinity of the exposed steel (i.e. steel won’t corrode until all the zinc has been sacrificed)^38^. In addition, the commercially available steel contain a standard commercial “Quality Electro Galvanized Steel Sheet” of which Zn is electrolytically coated at 300 A/m^2^ current density to protect it from corrosion; nevertheless might peel off at high current densities such as those applied in our experiments (60–100 A/m^2^)^39^. Consequently, it could be deduced that the sacrificial loss of Zn^2+^ ions in aqueous solution, released from the coated steel mesh electrodes, has generated smaller charged colloidal particles prior to the formation of cationic colloidal polyhydroxy Al^3+^ ions. Hence, slower rates of coagulation, due to Al^3+^, were observed within the first 30 min. These results are in good agreement with those presented in Fig. 4 (i.e. PSD showed no change at 30 min, yet has increased dramatically then after at all current densities). However, at higher current densities, the effect of ionic-electromigration has become clearly predominant over flocs-electrosettling, resulting in a significant reduction of Zn and Cu concentrations in the sludge suspension (biosolids) after 30 min. Another possibility to the initial increase of Zn concentration in the sludge suspension could be attributed the fact that Zn coated steel mesh inserted in an acidic solution (such as in our experiment with pH = 3) acts as a galvanic cell in the beginning through which extra Zn is released to the aqueous solution which may settle as ZnS (s) (i.e. the presence of S (27128.75 mg/kg) could have contributed to forming moderately insoluble ZnS at weak acidic conditions; Zn^2+^_(aq)_ + S^2−^_(aq)_ → ZnS_(s)_)^40^.

During this phase, the limited removal of Cu and Zn from the sludge was facilitated by electrochemically-induced hydrolysis, coagulation, flocculation of metals ions and colloidal particles through the formation of hydroxy and polyhydroxy derivatives of aluminum from the anode^6^. These aluminum hydroxide coagulants facilitated the formation of sludge flocs in the mixed liquor by producing denser flocs^41^,^42^. An increased current density also enhanced gas bubble generation at the electrodes which facilitates the removal of pollutants by floatation^15^,^16^. Due to electrostatic attraction, hydrated metal ions grow into larger and denser flocs which settled and precipitated at the bottom of the cell. However, some of these flocs remained less dense and light enough to be displaced upward by buoyancy force due to flotation. These flocs were collected at the surface of the cell. On the other hand, part of the charged metal ions were attracted to the oppositely charged electrodes (anode and cathode) through electrophoresis and deposited over the surface of electrodes^43^,^44^. Also, it was observed that an increase in current density and residence time resulted in increase in the mean PSD of sludge particles shown in Fig. 4a. However, there was no appreciable variation during the first 30 min. The current density of 100 A/m^2^ offered significant impact on mean PSD – an increase from 31 μm for the raw sludge to 38 μm for the electrokinetically treated sludge after 120 min. An increase in the sludge density resulting from the electrocoagulation of sludge particles was responsible for the increase in PSD as the applied current density increased. The PSD, which was also a measure of the degree of flocculation arising from EK treatment, contributed to the flocculation of colloidal sludge particles and dewaterability of the sludge. Filterability test (TTF) was used to assess the dewaterability of the electrokinetically treated sludge. It can be observed from Fig. 4b that the sludge TTF was reduced as residence time increased, regardless of the applied current density. However, when current density and residence time were increased together, more reduction in TTF of sludge slurry was observed. For example, after 120 min, TTF of 30 s was recorded at 100 A/m^2^ indicating 85.7% reduction. The sludge TTF was found to be closely related to the sludge PSD. The larger the sludge PSD, the higher is the tendency of the sludge to be dewatered and the lesser is the time taken to filter out the liquid portion of the mixed liquor. This infer that EK treatment has improved the release of bound water from the sludge by minimizing the time taken to coagulate the sludge^16^. At higher current density, the formation of more aluminum hydroxides in the mixed liquor ensured the promotion of alkaline conditions in the mixed liquor. Therefore, the mixed liquor pH was more alkaline at 100 A/m^2^ as compared to pH recorded at 20 and 60 A/m^2^ (Fig. 4c). The mixed liquor pH at 100 A/m^2^ increased from 6.91 at the start of the experiment to 8.27 after 120 min. For each applied current density, the mixed liquor pH increased with the residence time.

### Effect of chemical conditioners at optimized EK conditions

**Figure 5:**
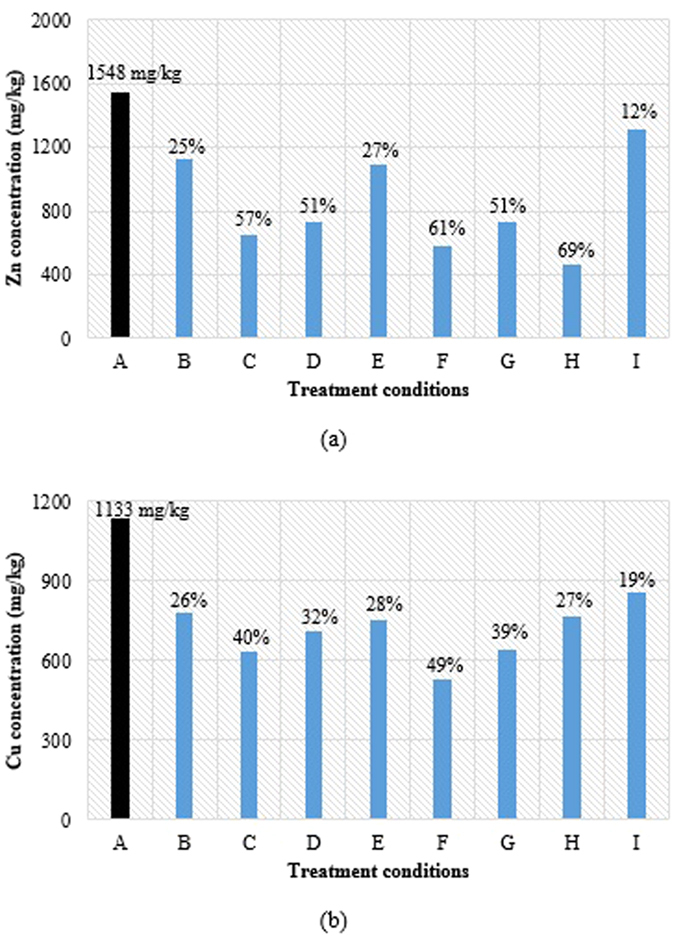
Impact of acidic conditions on (**a**) Zn and (**b**) Cu removal during EK (in terms of removal efficiency). **A:** Without EK treatment (raw secondary sludge). **B:** With EK treatment. **C:** With EK treatment and addition of HNO_3_ at pH 3. **D:** With EK treatment and addition of HNO_3_ at pH 4. **E:** With EK treatment and addition of HNO_3_ at pH 6–7. **F:** With EK treatment and addition of aqua regia at pH 3. **G:** With EK treatment and addition of EDTA + H_2_SO_4_ at pH 3. **H:** With EK treatment and addition of aqua regia + H_2_SO_4_ at pH 3.

After evaluating the findings reported in the optimization of EK conditions for heavy metal removal; an applied current density of 60 A/m^2^ and 120 min of residence time were selected as optimum conditions since they provided the highest removal of Cu and Zn. However, the application of 60 A/m^2^ on the initial sludge for 120 min only resulted in the drop of Cu from 1133 to 920 mg/kg (18.8% removal) and Zn from 1548 to 1332 mg/kg (13.95% removal). These removal efficiencies are still low and were further enhanced by boosting their solubility in the slurry suspension through the addition of chemical conditioners. For this acidified EK treatment, Cu and Zn were removed mainly from the foam layer (i.e. upward removal or electrofloatation). Heavy metal segregation in the foam layer dominated other mechanisms during the acidified EK treatment as a result of electroflotation arising from increased solubility. Thin layer deposits were also obtained on the electrodes surface. The variety of tested acidic additives at different low pH ranges is shown in Fig. 5. Experimental results presented in Fig. 5a show that aqua regia + H_2_SO_4_ at pH = 3 (*labeled as H*) gave the highest removal efficiencies of 69% for Zn. However, as shown in Fig. 5b, the highest Cu removal efficiency of 49% was achieved when aqua regia at pH = 3 (*labeled as F*) was used as chemical conditioner in the acidified EK treatment.

### Effect of aqua regia and residence time on removal efficiency

**Figure 6:**
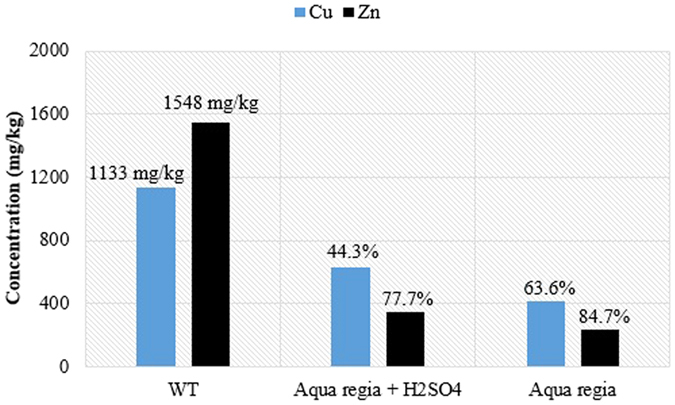
Cu and Zn concentration (in terms of removal efficiency) obtained from acidified EK treatment at residence time of 16 h at 60 A/m^2^. WT refers to raw sludge without treatment.

Under the optimized conditions of current density of 60 A/m^2^ and acidic conditioners (i.e. aqua regia at pH 3 for Cu, and aqua regia + H_2_SO_4_ at pH 3 for Zn); it is indeed desirable to understand the role of these conditioners for better removal of Zn and Cu from sludge slurry. The highest removal efficiencies reported during the optimization of EK conditions for heavy metal removal was obtained when the sludge residence time was 120 min. However, this residence time is too low and may not be effective for large-scale sludge treatment. Therefore, further experiments were performed to evaluate the impact of the chemical additives at longer time of treatment in order to mimic real life situations. The residence time was increased to 16 h. Meanwhile, this higher residence time was still very low (less than 1 day) when compared with the values used in many practical EK treatment of sewage sludge. An increase in the EK treatment time to 16 h resulted in further reductions in residual Cu and Zn in the sludge, as shown in Fig. 6.

It can be seen from Fig. 6 that the conditioning of the sludge with only aqua regia resulted in higher removal efficiencies of both Cu and Zn at a residence time of 16 h, when compared with the addition of aqua regia + H_2_SO_4_. This shows that the FNA in aqua regia has a more pronounced oxidizing or degradation capability than sulfuric acid. Removal efficiencies of 44.3 and 77.7% were obtained for Cu and Zn, respectively, when aqua regia + H_2_SO_4_ mixture was used as the conditioner; as compared to 63.6 and 84.7% recorded for Cu and Zn, respectively when only aqua regia was used.

Considering Zn as an example, the role of aqua regia can be explained through the following reactions (equations 1–3) that took place during the EK treatment:


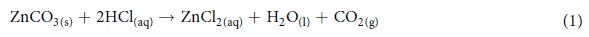

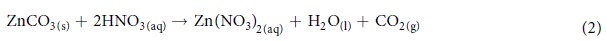


The lowest concentration of Cu and Zn in the treated sludge was observed at pH 3. The addition of acid to the wastewater secondary sludge during EK treatment facilitated the exchange of protons (from the acid) leading to the dissociative release of heavy metals in the mixed liquor, according to equation (3):


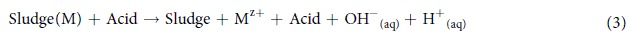


The results are in line with the general understanding of the electrochemical reactivity series of metals. Cu showed lower removal because it reacts slowly with dilute acids and preferably with concentrated nitric acid as compared to other metals (Zn) lying above hydrogen in the activity series. Hence, Zn can react more readily. The previous findings of our group^6^,^16^ revealed that heavy metals are found in urban waste sludge as metal carbonates, silicates, and aluminates which are bound within the metal salts lattice in the sludge. Acids can break the existing metal salt lattice structure and convert them into more readily available metal forms (oxides, sulfides, hydroxides, etc.). Those could be solubilized in aqueous phase and made available for electromigration by the digestion with strong acids and subsequently trapped in chelating agents through coordination complex^9^. Higher removal efficiency of Zn as compared to Cu can also be attributed to the more pronounced availability of ZnCl_2_, as compared to ZnSO_4_. The solubility of ZnCl_2_ is more favored in aqueous phase; therefore ZnCl_2_ is more available during upward removal than ZnSO_4_^45^ although ZnSO_4_ can further react with hypochlorous acid species found in aqua regia solution and yield ZnCl_2_. Sulfate salts of Zn, when present in any acidic solution containing HCl(aq), are more likely to undergo double displacement reaction shown in equation (4):


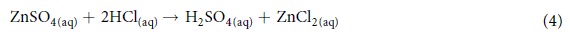


It has been reported that ZnCl_2_ is 7.48 times more readily soluble in aqueous phase than ZnSO_4_^45^. Thus, the production of ZnCl_2_ enhanced the possibility of upward lifting through gas bubbles as compared to ZnSO_4_. Two important observations which led to the removal of Cu and Zn were noted during acid conditioning: (1) formation of foam layer of flocs at the top of the reactor, and (2) deposition of sludge over the surface of electrodes in general and on the surface of the cathode in particular as reported earlier. In the electrolysis of water in the sludge, hydrogen cations (H^+^) move to the cathode to form hydrogen gas. Likewise, hydroxide ions (OH^−^) were displaced to the anode to yield oxygen gas upon oxidation. The formation of hydrogen and oxygen gas bubbles at the electrodes was particularly enhanced when acids were added. Since the objective of this process design was the removal of metals from the wastewater secondary sludge, the downward aggregation and settling in the sludge was undesirable. The upward removal of toxic metals is the most desired feature of this process design. These produced gas bubbles at the anode and cathode provided the buoyancy force that was greater than the gravitational (settling) force on the floc particles; hence the sludge particles were lifted up to form foam layer at the mixed liquor surface in the reactor. Apart from this, the gas bubbles reinforced the mixing in the liquor, thereby enhancing the electromigration of flocs and the horizontal deposition on the electrodes (i.e. electromigration of ions).

The reaction of Cu or Cu-bounded substances with nitric acid is based on the strength of acidic solution, whether diluted or concentrated. As nitric acid is a strong oxidizing agent, a higher oxidation state of nitrogen (IV) oxide is formed when the nitric acid is concentrated. The reaction may proceed as shown in equation (5):


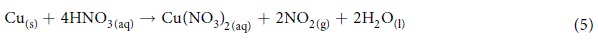


Moreover, when dilute nitric acid is considered (as the acid was added to 2 L of mixed liquor), the more probable process is shown in equation (6):


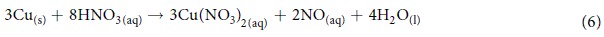


Aqua regia may also dissociate into volatile products - nitrosyl chloride and chlorine represented by fuming property and characteristic yellow color, as shown in equation (7). If these volatile products escape from the solution, the aqua regia may lose its potency and concentration:


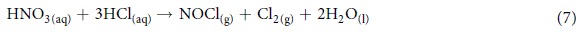


Nitrosyl chloride can further decompose into nitric oxide and chlorine in an equilibrium-limited dissociation equation (8):


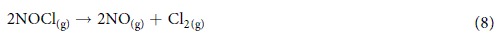


Therefore, a diversity of nitric oxide (NO), peroxy nitrous acid, peroxy nitrous oxide, and peroxy nitroso group are expected to be present in the mixed liquor. These fragments of nitrocyl group originating from aqua regia are responsible for the disruption of biogenic EPS and MLVSS^12^ and organic-inorganically bounded Zn and Cu species^6^,^15^. This disruption resulted into enhanced removal of exposed and oxidized metal ions. Minimum optimized use of aqua regia for greener, economical and efficient removal of Zn and Cu from wastewater secondary sludge has also been partially reported in a recent article^12^. Addition of metal nitrite to the mixed liquor resulted in 74% Zn and 49% Cu removal due to the presence of FNA. However, the actual modes of action of FNA and reaction mechanisms were not explained. Nitrates undergo a continuous chain of reduction proceeding through nitrites to nitric oxide and ending up with free nitrogen in the nitrogen cycle^46^. Moreover, under weak acidic environment of nitrite, formation of nitric oxide and other nitrogen oxide species would occur, and potent antimicrobial effects of these species against a broad range of potential pathogens could be observed^47^.

Transition metals such as Zn and Cu possess a special bonding association with NO group (nitrosyl) because of its ability to form metal nitrosyl complexes which can behave reversibly during acid–base equilibrium^48^. Two main types of nitrosyl complexes can exist: (1) linear (FTIR range 1650–1900 1/cm) and (2) bent (1525–1690 1/cm). The differing vibrational frequencies reflect the differing N-O bond orders for linear (triple bond) and bent NO (double bond). NO groups are isoelectronic with CO groups and thus can donate 3 electrons, producing a triple transition metal-nitrogen bond.

Furthermore, due to greater electronegativity of nitrogen than carbon, metal-nitrosyl complexes are more electrophilic as compared to its analogue metal carbonyl complexes. Hence nucleophiles can react with these metal nitrosyl complexes^48^. In addition, peroxynitrous acid (ONOOH) serves as a support for NO and it may also act as a nitrosating agent. Due to strong inductive effect of NO, the O-O bond got weaker as compared to that in hydrogen peroxide, considering the bond strengths of 90 and 170 kJ/mol^49^. Since ammonia is already present in the secondary sludge, ammonium can also be oxidized to a variety of nitrogen oxides, including nitrites (NO_2_^−^) and nitrates (NO_3_^−^). The cycle is completed by the serial reduction of nitrate to nitrite, nitric oxide, nitrous oxide, and finally, nitrogen gas^46^. It is thus evident that peroxynitroso group would contribute to the oxidative stress caused by the nitrocyle. Free radical NO specie in the mixed liquor would also react with O_2_, leading to the formation of peroxynitrite anion (ONOO) and activation of NO-dependent oxidative stress on biogenic species^50^. This stress would also be responsible for the disintegration of biological and organic species that host the bounded toxic metals. Therefore, the disintegration of metal binding species can be estimated through the decrease in sludge biogenic species due to nitric oxide and its peroxy derivatives in the aqua regia.

The physicochemical characteristics of the treated sludge at this stage were also investigated. Sludge properties such as PSD, TTF, SVI, MLSS, MLVSS, supernatant turbidity, and %TS were also determined. Changes in these physicochemical properties are shown in Table 5. It was observed that larger flocs were formed during the acidified EK treatment at residence time of 16 h. The mean PSD of sludge particles increased from 32.3 to 41 μm. However, this increase was not linear. The formation of larger flocs (55 μm) was observed at 8 h of treatment, after which a reduction in the floc size (41 μm) was reported after 16 h. The increase in the floc size to 55 μm after 8 h of treatment was mainly due to electrocoagulation activity whereby repulsive forces between the particles surfaces were reduced. However, a reduction in the floc size to 41 μm after 16 h of treatment was attributable to the removal of the bound water via electroosmosis phenomena^6^,^15^. Overall, the mean PSD of the initial sludge increased by 26.9% after 16 h of acidified EK treatment, showing that denser flocs were obtained.

Results in Table 5 show that the time needed to filter the sludge slurry (TTF) after treatment. The time needed to filter 100 mL of raw sludge sample was 120 s. However, after treatment, TTF decreased by 93.3%. EK treatment has been known to induce electroosmosis phenomenon. Electroosmosis is the outward movement of water from the mixed liquor as a result of the electrical gradient between the electrodes^15^. The application of voltage in the EK batch reactor released the physically bonded water out of the suspended solids, thereby improving the time needed to filter out the liquid portion. This means that EK treatment with aqua regia acidification at pH 3 produces denser flocs with good settling and filtering properties after 16 h of treatment at current density of 60 A/m^2^. The applied current density also reduced the SVI (Table 5) and improved sludge dewaterability by decreasing the specific resistance of the sludge to filtration. A 20.8% reduction in SVI from 120 to 95 mL/g was observed. Changes in the morphological and structural properties of the sludge particles were caused by the applied current, as evident in the sludge color change from dark to near-grey and mean PSD alteration.

Moreover, a slight rise in MLSS from 9560 to 9640 mg/L was observed. This slight increase resulted from the formation of coagulated Al^3+^ species in the mixed liquor due to the oxidative dissociation of ions from the aluminum anode. The increase in MLSS could have been greater but it was minimized by the deposition of coagulated sludge on the electrodes. However, a decrease in MLVSS (predominantly microbial disintegration fragments) was observed. This decrease in MLVSS can be explained by presence of NO and peroxy nitrous acid radicals as well as electrically induced inhibition in microbial growth^49^,^50^,^51^. A considerable reduction in the turbidity of supernatant obtained from the mixed liquor as a result of electrokinetically induced aggregation of smaller flocs and their subsequent displacement (in both vertical and horizontal directions), yielding a clear supernatant beneath the foam layer was also observed. Turbidity data of supernatant indicates that the supernatant obtained from this study is a potential resource for water recovery from sludge slurry under process. The 2 L sludge slurry produced 1 L of supernatant with a very low turbidity of 0.25 NTU. About 85% decrease in supernatant turbidity was achieved. The sludge slurry used in the present study was diluted and contained 0.90% as TS in raw. However, an increase in the TS to 1% was achieved after treatment.

### Post-treatment with pectin for sustainable recovery

**Figure 7:**
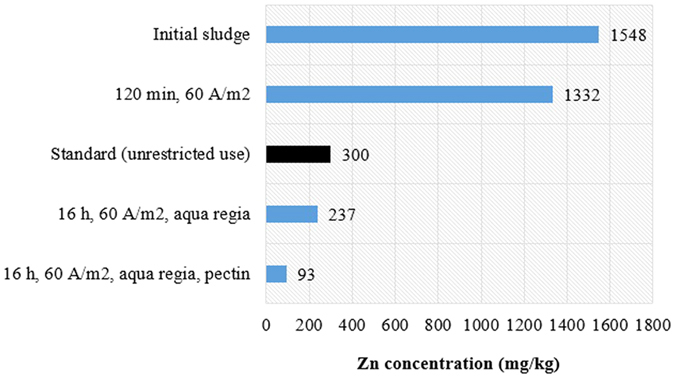
Zn concentration at different stages of treatment.

A further increase in current density or exposure of sludge to electric field can lead to a costly and less economically sustainable operation. Therefore, post-treatment using a cost-effective, green and organic biosorbent (pectin) has been carried out in order to further reduce the toxic heavy metal load in the sludge after acidified EK treatment. The extracted pectin enhanced coagulation and capturing of the targeted divalent metal cations and salts. Using Zn as the representative heavy metal here, the increase recorded for the removal efficiency of Zn from EK treatment without aqua regia to EK treatment with aqua regia and then to the post-treatment stage can be seen in Fig. 7.

This strategy has successfully eliminated Zn even below the prescribed standard for unrestricted use of biosolids locally and globally^52^. A current density of 60 A/m^2^ with aqua regia addition for 16 h and pectin post-treatment would significantly reduce Zn concentration in sewage sludge from 1548 to 93 mg/kg (below the minimum acceptable value of 300 mg/kg). Moreover, this work investigated the treatment of acidified EK with pectin on other multivalent ions and heavy metals indicating a great potential and possible recovery of many of those metals. Table 6 shows the final concentration of Mg, Ca, P, S, K, and Pb in the treated biosolids.

In order to investigate the economic viability of this strategy, a pilot scale experimental run of 16 L capacity was tested. This pilot scale showed excellent reproducibility of the technology. The findings obtained have paved the way for an industrial scale process design for high level of toxic heavy metal removal from biosolids. This proposed method offers very encouraging initial statistics on the environmental and economic costs. According to recent studies, sustainable management of City waste sludge gulps about 40 to 65% of the operational costs of wastewater treatment plants^7^,^53^. Power load and aluminum consumption are also essential cost factors during EK treatment. A previous study on EK treatment of sludge has reported a power load consumption of 8.9 kWh/m^3^ and aluminum consumption of 0.18 kg Al/m^3 ^8. Moreover, an overall operational cost of 0.86 USD/m^3^ has been reported for electrocoagulation unit operating at 100–150 A/m^2^ for 40 min. For sludge treated by only acidification and nitrite addition, the chemical costs were estimated to be 42.5 USD/ton (156 AED/ton) of dry sludge and 13.1 USD/wet ton (48 AED/wet ton)^12^. For this study, the cost components for the pilot experiment are shown in Table 7.

The sum of the operating cost components gave a total cost of 2.563 USD/m^3^ of wet sludge. This total operating cost translates to USD 1.972/wet ton (7.25 AED/wet ton). It is worth noting that a very small quantity of chemical has been used in the 16 L pilot unit (16 mL of aqua regia) to obtain about 94% of Zn. The operating cost of the unit was observed to be lower than previously reported values in studies that have employed other strategies^8^,^12^. Therefore, the cost analysis used in this study shows that the proposed treatment scheme is economically viable.

## Concluding Remarks

EK treatment under aqua regia condition has enhanced heavy metal removal (84.7 and 63.6% of Zn and Cu, respectively) from wastewater secondary sludge, much higher than the latest reported values of 49 and 74%^12^. The wastewater secondary sludge was obtained from the municipal wastewater treatment plant (MBR plant) at Masdar City, Abu Dhabi, United Arab Emirates. EK treatment under aqua regia condition at 60 A/m^2^ and 16 h generated denser settled solids (4 μm at SVI of 95 mL/g) with very low TTF (8 s) and clear sludge supernatant (0.25 NTU). This method applies waste-to-value concept of using biogenic chelating agent or pectin from citrus peels instead of continuous addition of aqua regia or anodic coagulants. Pectin as EK post-treatment agent enhanced the removal of Zn up to 93.7%. The quality of the generated biosolids met the standards and regulations in Abu Dhabi for *unrestricted use* of Zn and *controlled use* of Cu. Compared to landfilling and incineration, utilization of sludge for agricultural use is the best alternative for sludge disposal because it recycles both nutrients and organic matter once the heavy metals in sludge are reduced. An upward removal pattern (flotation) favors metal removal from biosolids. Supernatant water, which can be used as an additional source of water, is an added advantage of the proposed treatment method in this paper. Very small quantity of aqua regia was used to enhance the EK process, resulting in minimal operating cost. In future research studies, this study can be extended in two directions - one for water recovery and the other for application of treated sludge as soil conditioning agent (fertilizer). Currently, the treated sludge generated after treatment are being investigated for enhanced vegetation production in saline soil.

## Additional Information

**How to cite this article**: Hasan, S. W. *et al*. Molecular and ionic-scale chemical mechanisms behind the role of nitrocyl group in the electrochemical removal of heavy metals from sludge. *Sci. Rep.* **6**, 31828; doi: 10.1038/srep31828 (2016).

## Figures and Tables

**Table 1:** Initial properties of wastewater secondary sludge.

Property	Value	Unit
Cu	1133	mg/kg dry TS
Zn	1548	mg/kg dry TS
pH	7.7	—
Total solids (TS)	9170	mg/L
%TS	0.9	%
MLSS	8260	mg/L
MLVSS	6990	mg/L
Electrical conductivity (EC)	702	μS/cm
PSD	32.3	μm
TTF	120	S
SVI	120	mL/g

**Table 2:** Concentration of ions and heavy metals in raw wastewater secondary sludge (biosolids) and discharge limit standards (mg/kg) in Abu Dhabi^18^.

Metal	Concentration (mg/kg)	Regulatory standard (Unrestricted use*)	Regulatory standard (Controlled use**)
Al	1964.49	N/A	N/A
Mg	6169.41	N/A	N/A
Bal	859254	N/A	N/A
Si	23277.17	N/A	N/A
S	27128.75	N/A	N/A
P	22785.67	N/A	N/A
K	10962.49	N/A	N/A
Cl	6021.53	N/A	N/A
Ca	32894.1	N/A	N/A
Sc	150.11	N/A	N/A
Ti	3404.91	N/A	N/A
V	27.74	N/A	N/A
Cr	<LOD	400	1000
Mn	76.4	N/A	N/A
Fe	6971.36	N/A	N/A
Cu	1153.31	150	1000
Zn	1589.25	300	2500
Rb	30.27	N/A	N/A
Sr	194.69	N/A	N/A
Zr	21.2	N/A	N/A
Nb	3.92	N/A	N/A
Mo	16.13	20	75
Pd	5.21	N/A	N/A
Cd	16.74	N/A	N/A
Pb	9.68	N/A	N/A

^*^Unrestricted use involves frequent and uncontrolled exposure of the general public to biosolids.

^**^Controlled use involves infrequent and controlled public exposure to biosolids.

**Table 3:** Analytical methods for measurement of sludge physicochemical properties.

Sludge property	Analytical method
Cu, Zn	X-Ray Fluorescence (XRF) Analyzer
EC, pH, temperature, DO	HQ40d Multi DO Meter
TS, %TS. MLSS, MLVSS, TTF, SVI	APHA Standard Methods^54^
Mean particle size diameter (PSD)	HORIBA Laser Particle Scattering Analyzer

**Table 4:** Concentrations of chemical conditioners.

Chemical conditioner	Formula	Concentration
Sulfuric acid	H_2_SO_4_	95–97%
Nitric acid	HNO_3_	≥65%
Ethylene diamine tetra acetic acid	EDTA	M

**Table 5:** Change in physicochemical properties of treated sludge after 16 h at 60 A/m^2^ and pH 3 (via aqua regia).

	Initial sludge	After 16 h at 60 A/m^2^ and pH 3 (via aqua regia)	% Change
PSD (μm)	32.3	41	26.9% increase
TTF (s)	120	8.04	93.3% decrease
SVI (mL/g)	120	95	20.8% decrease
MLVSS	6990	4500	35.6% decrease
MLSS	8260	9640	16.7% increase
Supernatant turbidity	1.71	0.25	85.4% decrease
%TS	0.9	1	11.1% increase

**Table 6:** Concentration (mg/kg) of ions and heavy metals in treated wastewater secondary sludge (biosolids) after acidified (with aqua regia) EK (at 16 h at 60 A/m^2^) and pectin treatment.

Metal	Concentratio (mg/kg)	Regulatory standard (Unrestricted use*)	Regulatory standard (Controlled use**)
Mg	<LOD	N/A	N/A
S	21925.98	N/A	N/A
P	12937.61	N/A	N/A
K	822.31	N/A	N/A
Ca	1362.94	N/A	N/A
Sc	11.55	N/A	N/A
Cr	<LOD	400	1000
Mn	<LOD	N/A	N/A
Fe	5547.46	N/A	N/A
Cu	374.14	150	1000
Zn	93.35	300	2500
Rb	26.18	N/A	N/A
Sr	14.61	N/A	N/A
Cd	12.24	N/A	N/A
Pb	<LOD	N/A	N/A

**Table 7:** Operating cost components for the treatment of 16 L of wastewater secondary sludge.

Items	Values	Unit
Used aqua regia	16	mL/L
Aluminum consumed	0.058	mg/L
Electrical energy consumed	6.45	Wh/L
Cost of aqua regia cost	0.16	USD/L
Cost of aluminum consumed	1.85	USD/kg
Cost of electric energy consumed	0.03	USD/kWh
